# Osteoporosis and Alzheimer’s Disease: The Role of Sclerostin

**DOI:** 10.1007/s11914-026-00993-y

**Published:** 2026-10-21

**Authors:** Lorelé Gagiano, Ziming Chen, William G. Blakeney, Christopher Jones, Frank Mastaglia, Minghao Zheng, Jun Yuan

**Affiliations:** 1 School of Biomedical Sciences, The University of Western Australia, 35 Stirling Highway, Perth, WA 6009 Australia; 2 Centre for Translational Orthopaedic Research, School of Biomedical Sciences, The University of Western Australia, 35 Stirling Highway, Perth, WA 6009 Australia; 3 Department of Orthopaedic Surgery, Royal Perth Hospital, Wellington Street, Perth, WA 6000 Australia; 4 Department of Surgery, Medical School, The University of Western Australia, 35 Stirling Highway, Perth, WA 6009 Australia; 5 Department of Orthopaedic Surgery, Fremantle and Fiona Stanley Hospitals, Perth, WA 6150 Australia; 6 Department of Orthopaedic Surgery, The Orthopaedic Research Foundation of Western Australia, Perth, WA 6010 Australia; 7 Division of Surgery, The University of Western Australia, Perth, WA 6009 Australia; 8 Perron Institute for Neurological and Translational Science, The University of Western Australia, 8 Verdun St, Nedlands, WA 6009 Australia

**Keywords:** Osteoporosis, Alzheimer’s disease, Sclerostin, Wnt/β-catenin, Aging, Bone-brain axis

## Abstract

**Purpose of Review:**

Little is known about the underlying cause contributing to the association between Osteoporosis (OP) and Alzheimer’s disease (AD). This review highlights the emerging role of sclerostin, a protein mainly produced by osteocytes, in the newly discovered crosstalk between bone and brain, with a focus on its potential contribution to the relationship between OP and AD.

**Recent Findings:**

Circulating sclerostin levels increase with aging and are positively associated with brain amyloid-β burden in older adults. Studies demonstrate that elevated sclerostin can cross the blood-brain barrier, promote amyloid-β peptide deposition, and impair synaptic plasticity by suppressing Wnt/β-catenin signalling, whereas sclerostin inhibition restores neuronal plasticity and attenuates AD pathology.

**Summary:**

Sclerostin represents a promising target in the bone-brain interaction pathway. Its dual role as a measurable circulating biomarker and a modifiable component offers new opportunities for early risk assessment and therapeutic repurposing. Future longitudinal and interventional studies are essential to confirm the association between sclerostin and risk of AD, and to clarify whether targeting sclerostin can delay the onset of the disease or modify its trajectory.

## Introduction

Osteoporosis (OP) and Alzheimer’s disease (AD) represent two of the most prevalent age-related disorders, each imposing substantial healthcare burdens and profoundly reducing quality of life for elderly people worldwide [1–5]. AD is the most common cause of dementia and is characterised by progressive neocortical degeneration leading to severe cognitive and functional decline. OP is defined by reduced bone mineral density (BMD) and impaired bone microarchitecture, resulting in skeletal fragility and increased fracture risk [5, 6]. Although long regarded as two distinct clinical entities, a compelling and growing body of evidence points to a complex and potentially bidirectional relationship between the two disorders. Rather than reflecting coincidence or aging alone, their overlap appears to be driven by shared risk factors, such as low physical activity, vitamin D deficiency and sarcopenia [7, 8]. Moreover, both conditions emerge within a milieu of chronic inflammation, oxidative stress, vascular dysfunction, and impaired Wnt/β-catenin signalling, which are the mechanisms that simultaneously compromise skeletal integrity and accelerate neurodegeneration [9–11]. Notably, recent longitudinal studies suggest that individuals with OP are at increased risk of cognitive decline and dementia later in life [12, 13], particularly in women, underscoring the possibility of a shared underlying pathophysiology.

Beyond the traditional structural role, bone is now increasingly recognised as a dynamic endocrine organ that secretes multiple signalling molecules with effects extending far beyond the skeleton [14–16]. For instance, osteocalcin (OCN) and lipocalin 2 (LCN2) secreted by bone cells have been shown by in vivo studies to exert distinct influences on central nervous system function, including brain development, age-related cognitive decline [17, 18] and appetite regulation [19]. This expanding repertoire of bone-derived signals positions the bone as a potential hub and support the concept of a “bone-brain axis”, in which bone-derived signals contribute to inter-organ communication and actively regulate neural function and cognitive health [20–22]. While OCN and LCN2 have been relatively well characterised in this context, the role of other bone-derived factors remains poorly understood, highlighting an important knowledge gap.

Sclerostin, secreted primarily by osteocytes, is a potent inhibitor of the Wnt/β-catenin signalling pathway and a central regulator of bone formation [23–26]. Emerging evidence suggests that sclerostin may represent a mechanistic link between the OP and AD association, with potential roles in the pathophysiology of both bone fragility and neurodegeneration [14]. This review synthesises current evidence supporting the role of the bone-brain axis in the association between OP and AD, with a particular focus on sclerostin as a mediator of bone-brain crosstalk, and discusses its potential as both a biomarker of dementia risk and a therapeutic target at the interface of skeletal and cognitive health.

## Bone as a Signalling Hub Influencing Brain Function

Traditionally viewed as a passive structural framework for locomotion and mineral storage, bone is now recognised as a dynamic endocrine organ with systemic influence, including effects on the brain [20–22]. Osteoblasts and osteocytes secrete a variety of signalling molecules that act hormonally to regulate physiological processes such as glucose metabolism, appetite and neuronal activity [19, 27–30]. This shift has led to the concept of the bone-brain axis, highlighting evidence that bone-derived factors can modulate central nervous system function. Among the best-characterised bone-derived hormones are OCN and LCN2, both implicated in the regulation of brain function in vivo.

OCN, also called bone γ-carboxyglutamic acid (Gla) protein or BGP, was originally identified as a vitamin K-dependent, non-collagenous protein produced by osteoblasts and incorporated into the mineralised bone matrix, where its γ-carboxylated form binds hydroxyapatite [31–33]. Although initially considered primarily a bone matrix protein and subsequently widely used as a marker of osteoblast activity and bone formation, later studies revealed that OCN can enter the circulatory system [34]. In particular, circulating undercarboxylated OCN has been proposed as its hormonally active form [35]. In vivo studies have shown that circulating undercarboxylated OCN can cross the blood-brain barrier (BBB) and bind to the hippocampal receptor GPR158. This signalling regulates anxiety and cognition and has been linked to age-related cognitive decline [17]. Recent work further demonstrated that OCN ameliorates cognitive deficits in APP/PS1 transgenic AD mice by reducing amyloid-β (Aβ) accumulation, the deposition of which in the brain is a major pathological change of AD, and enhancing glycolysis in neuroglia [36]. Meanwhile, LCN2 was originally identified as a 25-kDa protein associated with human neutrophil granules and was therefore termed neutrophil gelatinase-associated lipocalin (NGAL) [37]. Functionally, LCN2 was first characterised as an innate immune factor that mediates iron homeostasis, specifically restricting pathogen growth by binding and sequestering iron-laden bacterial siderophores [38, 39]. It was subsequently found to be highly expressed and secreted by osteoblasts, leading to its recognition as a bone-derived endocrine factor [19]. Bone-derived LCN2 can cross the BBB and engage hypothalamic melanocortin-4 receptors (MC4R) to modulate feeding behaviour [19]. Animal studies suggest that LCN2 can also influence hippocampal signalling [40] and regulate Aβ dynamics [41, 42], potentially contributing to early AD-like pathology [43].

While animal studies consistently demonstrate that bone-derived OCN and LCN2 regulate brain function, evidence from human studies remains limited and inconsistent. Findings on OCN vary across cohorts, with both positive and null associations reported, which may be attributed to differences in measurement methods and participant characteristics [44]. Similarly, a meta-analysis investigating the role of LCN2 in AD revealed variability across biofluids: serum and plasma levels differed between AD and controls depending on age, whereas cerebrospinal fluid (CSF) levels showed no group differences. Nonetheless, increased LCN2 expression has been observed in AD-related brain regions, suggesting a role in disease pathophysiology [45]. In addition to these findings supporting the broader concept that bone-derived proteins can influence brain function and correlate with AD risk, emerging evidence points to an additional bone-secreted factor, sclerostin, that may provide critical mechanistic links between aging-related bone loss and cognitive impairment (discussed later). These mechanistic insights are reinforced by epidemiological observations revealing that structural and metabolic deterioration of bone closely parallels cognitive decline during aging.

## Association Between Osteoporosis and Alzheimer’s Disease: Insights from Observational Studies

An increasing volume of research uniformly points to a significant and bidirectional association between OP and AD. Epidemiological studies consistently report that individuals with AD exhibit lower BMD and a higher prevalence of OP [46–48], while those with reduced BMD or a history of fractures face an increased risk of cognitive decline and dementia, including AD [12, 49–52]. For example, a cohort study involving 157,988 participants reported a 1.49-fold higher prevalence of AD among individuals with OP compared to age- and sex-matched controls, suggesting that OP may contribute to increased AD susceptibility in adults over 40 years [51]. Similarly, a prospective cohort of 936 individuals followed up for five years demonstrated that those with BMD in the lowest quartile had a two-fold increased risk of transitioning to AD relative to those with higher BMD levels [52]. Notably, such associations have been observed not only for BMD measured by dual-energy X-ray absorptiometry (DXA), the gold-standard imaging modality, but also for heel BMD assessed by quantitative ultrasound (QUS) [53].

Further evidence comes from the Rotterdam Study, which assessed BMD in 3,651 dementia-free individuals aged 70 years and older, followed for a mean of 11 years. During follow-up, 688 participants (19%) developed dementia. Dementia incidence was significantly higher in the lowest BMD quartile compared with the highest, even after adjusting for age, sex, and family history of dementia. Specifically, participants with the lowest BMD were 42% more likely to develop dementia compared to those with the highest BMD [12]. Complementing these findings, the Canadian Multicentre Osteoporosis Study followed more than 2,300 older adults (≥ 65 years) for up to 16 years and found that cognitive decline was significantly associated with accelerated bone loss in women, independent of aging and other confounders. Approximately 13% of participants experienced clinically meaningful cognitive decline within five years, which was further linked to an increased risk of fractures in the subsequent decade. These findings provide longitudinal evidence that cognitive deterioration and skeletal fragility are interconnected processes, particularly in women [13].

Beyond epidemiological reports, genetic studies provide complementary insights. Recent Mendelian randomisation (MR) analyses suggest that the observed association between OP and AD may not reflect a direct causal relationship. A two-sample MR study by Hu et al. [54], using genome-wide association study (GWAS) datasets, tested the bidirectional relationship between OP and AD and found no evidence of significant causal effects in either direction after multiple sensitivity analyses. This divergence from cohort findings may reflect limitations of observational designs, including residual confounding and heterogeneous definitions of OP. Importantly, the MR evidence supports the interpretation that OP and AD likely share overlapping biological pathways rather than one disorder directly initiating the other.

Notably, the strength of the association between low BMD and dementia risk appears to attenuate with longer follow-up, suggesting that low BMD may emerge as a prodromal feature of dementia rather than a primary cause, potentially reflecting early systemic changes associated with toxic protein accumulation in the brain [12]. Together, the persistent and unexplained associations between bone loss and cognitive decline raise the possibility of underlying biological mechanisms linking skeletal fragility and neurodegeneration.

## Sclerostin: An Emerging Game Player in Bone-Brain Crosstalk

Sclerostin, predominantly produced by mature osteocytes, functions as a potent inhibitor of Wnt/β-catenin signalling [24]. Alterations in this pathway have been implicated not only in impaired bone formation and bone loss, but also in the neuropathology of AD [55]. Supporting this link, a recent network analysis to human bone and brain transcriptomes identified Wnt signalling as one of the most enriched molecular pathways shared between OP and AD [56]. Given the central role of sclerostin in modulating Wnt signalling, it therefore represents a compelling candidate for involvement in both skeletal fragility and cognitive dysfunction.

Encoded by the *SOST* gene, sclerostin exerts its effects on bone formation primarily by antagonising the canonical Wnt/β-catenin signalling pathway, which is essential for osteoblast proliferation, differentiation, and function [57–59]. Mechanistically, sclerostin binds to the Wnt co-receptors low-density lipoprotein receptor-related proteins 5 and 6 (LRP5/6), preventing Wnt ligands from engaging the Wnt-Frizzled-LRP complex and initiating downstream signalling [25, 60]. This blockade interrupts the phosphorylation cascade that normally releases β-catenin from its cytoplasmic destruction complex, thereby promoting β-catenin degradation and reducing T cell factor/lymphoid enhancer factor (TCF/LEF)-mediated transcription of osteogenic target genes [60]. As a result, bone formation is suppressed and resorption relatively enhanced, maintaining skeletal homeostasis by preventing excessive bone accrual.

The production of sclerostin in bone is tightly regulated by a combination of genetic, epigenetic, hormonal, and mechanical factors. Genetic polymorphisms within the *SOST* locus can influence circulating sclerostin levels, while DNA methylation of CpG islands in its promoter modulates transcriptional activity [61]. Hormonal regulation is equally critical: parathyroid hormone (PTH) potently suppresses sclerostin expression, whereas estrogen deficiency, as seen in postmenopausal OP, is associated with elevated sclerostin levels [62, 63]. Mechanical loading, such as weight-bearing exercise, also downregulates sclerostin, reflecting its role in mechanotransduction, whereby osteocytes sense strain and adjust bone remodelling accordingly [64]. Importantly, circulating sclerostin levels increase with aging [65]. This age-related elevation has been implicated not only in reduced bone formation but may also contribute to extra-skeletal processes, such as the age-associated cognitive decline.

### Sclerostin and Brain Aβ Deposition: Evidence from Human Studies

Abnormal accumulation of Aβ peptide in the brain is a hallmark pathological feature of AD, and it can begin years before symptom onset [66]. To investigate the potential link between sclerostin and AD, our group previously studied a cohort of 100 cognitively unimpaired older adults, in whom brain Aβ burden was assessed using positron emission tomography (PET) imaging [67]. Plasma sclerostin levels were found to be significantly higher in participants with abnormally high brain Aβ load, compared to those with normal levels of Aβ deposition. Moreover, Spearman’s correlation analysis showed that plasma sclerostin concentrations were positively associated with brain Aβ burden, indicating that higher systemic sclerostin may be an early biomarker or risk factor for AD-related pathology.

More recent studies have extended these findings to CSF. Elevated CSF sclerostin levels have been reported in patients with AD and were associated with established AD biomarkers, including Aβ42, total and phosphorylated tau, as well as dementia severity [68]. Furthermore, higher CSF sclerostin levels were associated with poorer global and domain-specific cognitive performance across the AD continuum, with particularly pronounced associations observed in individuals with mild cognitive impairments [69]. Together with the plasma and PET findings, these observations provide converging human evidence linking altered sclerostin levels with AD-related pathology and cognitive impairment, with biological plausibility supported by the established roles of Wnt/β-catenin signalling in the central nervous system.

### Sclerostin and AD Pathogenesis: The Molecular Mechanisms

Canonical Wnt/β-catenin signalling is known to be essential for neuronal differentiation, synaptic plasticity, and memory formation, and its disruption has been linked to neurodegeneration [70–72]. Studies involving animal models of AD consistently demonstrate Wnt pathway impairment [55, 73–75]. In particular, elevated levels of Wnt inhibitors such as Dickkopf-1 (Dkk1) are detected in the brains AD animal models, where they drive synaptic degeneration and impair long-term potentiation [74, 76–78]. Conversely, activation of Wnt ligands such as Wnt-3a and Wnt-7a enhance synapse formation, dendritic spine growth, and hippocampal neurogenesis, processes fundamental to learning and memory [71]. At the molecular level, reductions in β-catenin and increased glycogen synthase kinase-3β (GSK-3β) activity have been observed in AD pathogeneisis, mechanistically linking Wnt disruption to tau hyperphosphorylation and Aβ-induced toxicity [70, 71].

**Fig. 1:**
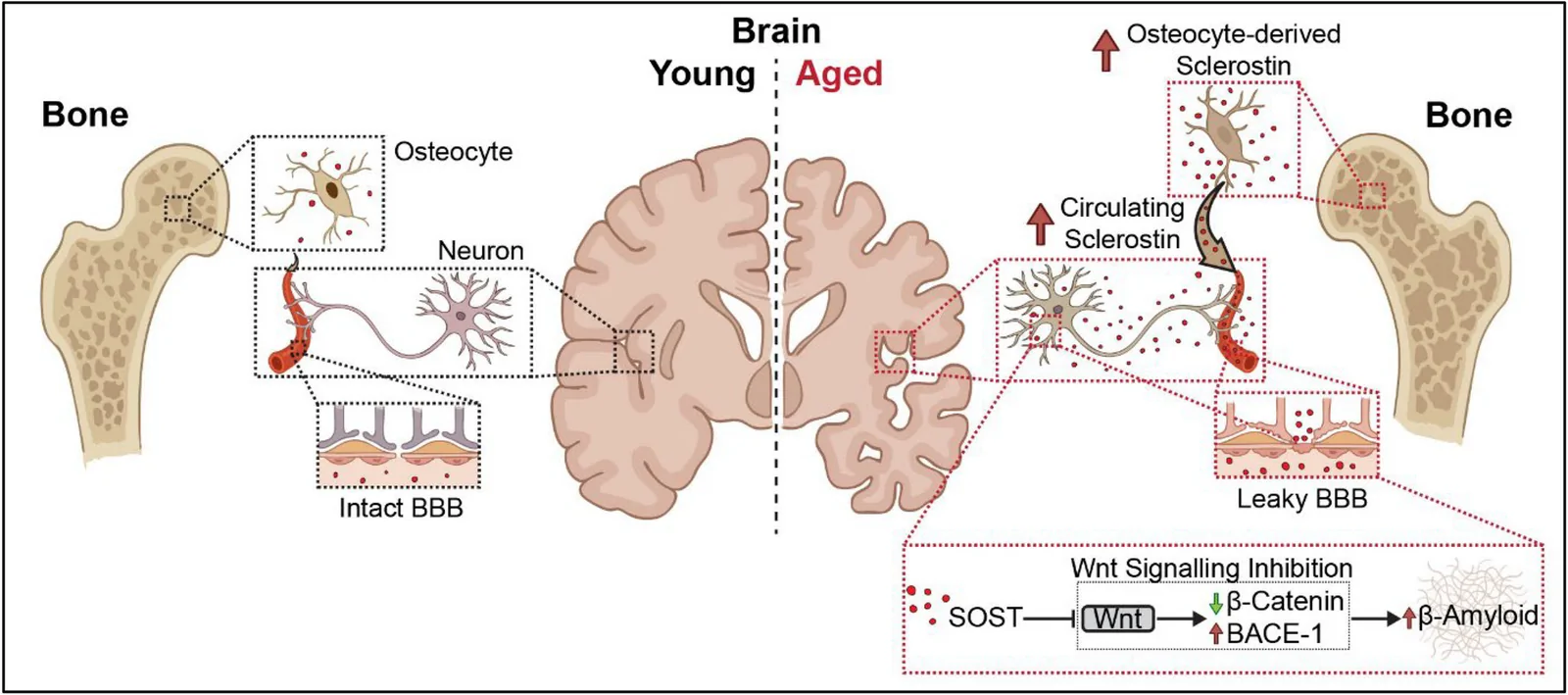
Potential role of sclerostin (SOST) in the pathogenesis of AD. During aging, osteocytes produce increased levels of sclerostin, which is released into the circulation. Elevated circulating sclerostin can cross the blood–brain barrier (BBB) and contribute to AD pathology by promoting amyloid-β (Aβ) production. Mechanistically, sclerostin upregulates β-catenin-dependent β-secretase 1 (BACE1) expression while reducing overall β-catenin levels, thereby enhancing Aβ generation

Complementary in vivo work has provided mechanistic insight into how sclerostin regulates cognition [79]. Shi et al. showed that osteocyte-derived sclerostin could cross the BBB in aged mice, and bone-specific overexpression of sclerostin suppressed Wnt/β-catenin signalling in the brain, resulting in impairment of synaptic plasticity and memory in aged mice of both sexes. Mechanistically, sclerostin promotes Aβ production through upregulating β-catenin-β-secretase 1 (BACE1) expression and decreasing β-catenin levels. Consistent with these findings, Shi et al. reported that in patients with AD, those with high serum sclerostin levels exhibited more severe cognitive impairment than those with lower serum sclerostin levels. Collectively, therefore, evidence from the clinical cohort, animal models, and mechanistic studies positions sclerostin as a novel contributor to AD pathogenesis and highlights its potential as a therapeutic target at the intersection of bone and brain health (Fig. 1).

### Bone-Brain Crosstalk: How does Sclerostin Cross the BBB?

The BBB is a selective semi-permeable membrane between the blood and the brain parenchyma. By regulating the passage of molecules, ions, and cells, cerebral blood vessels protect neural tissue from harmful substances while maintaining central homeostasis [80]. Despite human cohort and animal experimental evidence linking peripheral sclerostin levels with brain pathology and cognitive decline, the mechanism by which bone-derived sclerostin enters the brain remains unresolved. Although Shi et al. [79] recently demonstrated that circulating sclerostin can cross an intact BBB and enter the brain parenchyma under physiological conditions during aging, the specific transport pathway has yet to be defined.

From a biochemical perspective, sclerostin’s properties are not favourable for passive diffusion across the BBB. According to the computation analysis performed using ProtParam [81], sclerostin is a 22.5 kDa secreted glycoprotein with a theoretical isoelectric point (pI) of 9.43 (Table 1). Under physiological pH (~ 7.4), it carries a net positive charge, a feature that may influence its interaction with cell-surface receptors and potential passage across the BBB. By contrast, molecules that freely diffuse across BBB are typically small (< 450 Da), lipophilic, and often neutral or only weakly charged [82]. Thus, if bone-derived sclerostin does reach brain parenchyma, plausible routes include receptor‐mediated transcytosis, adsorptive transcytosis, carriage via extracellular vesicles (e.g. exosomes), or passage through a compromised BBB under aging or pathological conditions.

**Table 1:** Predicted physicochemical properties of sclerostin (ProtParam analysis)

Property	Value	Interpretation
Amino acids	213	Medium-sized protein
Molecular weight	24.0 kDa	Consistent with a secreted glycoprotein
Theoretical pI^*^	9.43	Positively charged at physiological pH
Net charged residues (R + K vs. D + E)^#^	35 vs. 23	Net positive
Instability index	36.96	Stable
Aliphatic index	64.2	Moderately thermostable
GRAVY score^§^	–0.733	Hydrophilic

^*^pI: isoelectric point

^#^Net charged residues: the overall positive or negative charge of a protein or peptide, calculated as the difference between positively charged (R + K) and negatively charged (D + E) amino acids. R, arginine; K, lysine; D, aspartic acid; E, glutamic acid

^§^GRAVY score: Grand Average of Hydropathy score, representing the average hydrophobicity of a protein, calculated from the hydropathy values of all amino acids. Positive values indicate hydrophobic, negative values indicate hydrophilic character

Mechanistic considerations support the potential for receptor-mediated pathways. Sclerostin binds to the Wnt co-receptors LRP5/6, which are expressed not only in osteocytes but also in brain endothelial cells and other components of the neurovascular unit. Endothelial Wnt/LRP5/6 signalling is essential for BBB development, integrity, and function [83]. This shared receptor system raises the possibility that sclerostin could interact with brain endothelial LRP receptors, either facilitating its transcytosis or indirectly modulating BBB properties. Nevertheless, no study has yet definitively demonstrated receptor-mediated transport of sclerostin across the BBB.

In sum, while peripheral sclerostin is correlated with aging, cognitive decline, and AD-related pathology, and substantial evidence supports that Wnt signalling pathways are responsive to sclerostin when centrally present, the hypothesis that bone-derived sclerostin freely traverses an intact BBB under basal conditions remains to be definitively proven in humans. Addressing this will require integrating human CSF/plasma biomarker studies, in vivo tracer biodistribution, in vitro BBB transport assays, and assessments of BBB permeability in aging and disease. Such multi‐modal evidence is essential for clarifying whether sclerostin acts as a bone-brain signalling factor via direct brain entry, or whether its impact is mediated via systemic or vascular‐endothelial mechanisms.

## Prospective Role of Sclerostin in AD Diagnosis and Treatment

Evidence from both cohort and animal studies suggests that sclerostin contributes to AD-related pathology. However, large-scale human data directly linking sclerostin to cognitive function and incident AD remain limited. Robust longitudinal studies will therefore be essential to confirm associations between circulating sclerostin and AD risk. Building on this foundation, sclerostin thereby offers two complementary avenues for advancing AD diagnosis and treatment.

### Sclerostin as a Biomarker

Circulating sclerostin holds promise as a blood-based biomarker for early risk stratification. Identifying biomarkers that reliably predict cognitive decline or progression to AD remains a critical unmet need. Our cohort study demonstrated that the addition of sclerostin to a panel of other established plasma biomarkers, including Aβ40, Aβ42, neurofilament light (NFL), glial fibrillary acidic protein (GFAP), total tau (t-tau), phosphorylated tau (p-tau181 and p-tau231), and especially the Aβ42/Aβ40 ratio, improved the accuracy of distinguishing older adults at risk for AD, defined by abnormal brain Aβ deposition (pre-AD status) [67]. As a stable and biologically relevant mediator linking bone and brain metabolism, sclerostin could therefore enable earlier identification of at-risk individuals and guide patient selection for preventive strategies or clinical trials.

### Sclerostin as a Therapeutic Target

Targeting sclerostin represents a promising strategy for slowing AD progression. Loss-of-function mutations in the *SOST* gene are known to cause rare high bone mass disorders, such as sclerosteosis and van Buchem disease [84]. This biology underpins therapeutic strategies that transiently block sclerostin. Romosozumab, a monoclonal anti-sclerostin antibody, is FDA-approved for treating postmenopausal OP by increasing bone formation, reducing resorption, and substantially lowering fracture risk [62, 85]. Preclinical studies demonstrate that sclerostin inhibition restores Wnt/β-catenin signalling, preserves synaptic integrity, and attenuates amyloidogenic processing in AD models [79]. Thus, romosozumab may hold dual potential for bone regeneration and neuroprotection, particularly through enhancing synaptic plasticity and microglial function by suppressing the activity of sclerostin.

Beyond AD-specific models, pharmacoepidemiologic evidence suggests broader neuroprotective effects of sclerostin inhibition. In a nationwide cohort, romosozumab use was associated with a significantly reduced risk of Parkinson’s disease compared with PTH receptor agonists, especially among women and individuals aged ≥ 75 years [86]. Given that cognitive dysfunction is a late-stage feature of PD, these findings suggest that Wnt/β-catenin activation via sclerostin inhibition may delay neurodegeneration more broadly. A logical next step is to investigate whether OP patients receiving romosozumab show altered incidence of AD or dementia compared with matched untreated populations, which can provide critical insight into its feasibility as a preventive therapy.

Despite its promise, the development of therapies targeting sclerostin faces several challenges. First, systemic activation of Wnt/β-catenin signalling carries risks, including aberrant bone overgrowth [87] and potential tumorigenesis [88, 89], underscoring the importance of careful dose optimisation and treatment duration. Second, it remains unclear whether peripherally administered anti-sclerostin therapies can sufficiently modulate central Wnt activity, given uncertainties about sclerostin transport across the BBB. Third, patient heterogeneity, such as differences in *APOE* genotype, sex, or baseline bone health, may influence therapeutic efficacy, necessitating precision-medicine approaches. Addressing these challenges through rigorous preclinical testing and carefully designed early-phase trials will be critical before translating sclerostin-targeted interventions to clinical practice.

Looking forward, advancing our understanding of sclerostin’s role in AD will require an integrated research agenda that spans population-based cohorts, biomarker testify, and therapeutic exploration. Future work should prioritise longitudinal studies in at-risk groups (e.g., older adults, *APOE* ε4 carriers, or individuals with mild cognitive impairment), mechanistic exploration of sclerostin’s central versus peripheral actions, and early-phase clinical trials of anti-sclerostin therapies in dementia.

## Conclusion

Sclerostin is emerging as a pivotal link between skeletal health and neurodegeneration, providing novel insight into how bone-derived signals may influence AD and related dementias. Experimental models strongly implicate sclerostin in the regulation of Wnt/β-catenin signalling, amyloidogenic processing, and synaptic plasticity, whereas human studies are only beginning to clarify its association with cognitive decline and AD. As a measurable circulating factor and the therapeutic target of approved osteoporosis drugs, sclerostin represents both a promising biomarker of cognitive ageing and a modifiable pathway in dementia. Establishing this connection would not only reposition osteoporosis therapies within the field of AD research, but also open a new frontier in understanding how skeletal signals shape brain aging and neurodegeneration.

## Key References

- Oury F, Khrimian L, Denny CA, Gardin A, Chamouni A, Goeden N, et al. Maternal and offspring pools of osteocalcin influence brain development and functions. Cell 2013;155:228–41. https://doi.org/doi.org/10.1016/j.cell.2013.08.042.
  - ⚬ This study provides the first in vivo evidence that bone-derived protein, osteocalcin, is necessary for brain development and cognition.
- Nagarajan A, Laird J, Ugochukwu O, Reppe S, Gautvik K, Ross RD, et al. Network Analysis of Brain and Bone Tissue Transcripts Reveals Shared Molecular Mechanisms Underlying Alzheimer’s Disease and Related Dementias and Osteoporosis. The Journals of Gerontology: Series A 2024;79:211. https://doi.org/10.1093/GERONA/GLAE211.
  - ⚬ This study provides evidence that Wnt signalling is one of the most enriched molecular pathways shared between OP and AD and related dementias, indicating the potential involvement of sclerostin as an inhibitor of Wnt signalling in the association of these two diseases.
- Yuan J, Pedrini S, Thota R, Doecke J, Chatterjee P, Sohrabi HR, et al. Elevated plasma sclerostin is associated with high brain amyloid-β load in cognitively normal older adults. NPJ Aging 2023;9.https://doi.org/10.1038/s41514-023-00114-4.
  - ⚬ This study provides the first human evidence linking a bone-derived protein to cerebral amyloid accumulation, underscoring the emerging concept of bone-brain crosstalk in neurodegenerative disease. It was found that elevated plasma sclerostin correlates with higher brain amyloid-β deposition in cognitively normal older adults.
- Shi T, Shen S, Shi Y, Wang Q, Zhang G, Lin J, et al. Osteocyte-derived sclerostin impairs cognitive function during ageing and Alzheimer’s disease progression. Nat Metab 2024;6:531–49. https://doi.org/10.1038/s42255-024-00989-x.
  - ⚬ This study shows that osteocyte-derived sclerostin can cross the BBB and demonstrates the molecular mechanism by which sclerostin promotes amyloid-β production, and accelerates cognitive decline in AD mouse models.
- Dicarlo M, Pignataro P, Urso D, Zecca C, Dell'Abate MT, Borlizzi F, et al. Cerebrospinal fluid sclerostin levels in the early Alzheimer's disease stages. Alzheimer's & Dementia: Diagnosis, Assessment& Disease Monitoring 2026;18:e70297. https://doi.org/10.1002/dad2.70297.
  - ⚬ This study demonstrated that CSF sclerostin rises with AD progression and is linked to core AD pathology, including reduced Aβ42 and increased tau, positioning it as a candidate biomarker for early disease detection.
- Pignataro P, Dicarlo M, Urso D, Zecca C, Colaianni G, Dell'Abate MT, et al. Elevated sclerostin levels in cerebrospinal fluid are associated with cognitive impairment in the Alzheimer's disease continuum. Alzheimer's & Dementia: Diagnosis, Assessment & Disease Monitoring 2026;18:e70417. https://doi.org/10.1002/dad2.70417.
  - ⚬ This study was the first to examine sclerostin's association with specific cognitive domains across the AD continuum, showing that elevated CSF sclerostin was linked to poorer performance in all affected domains, most notably in individuals with mild cognitive impairment.

## Data Availability

No datasets were generated or analysed during the current study.

## Declarations

### Prior publication

All figures and tables are original and never published before.

## References

[1] Rosen CJ. The epidemiology and pathogenesis of osteoporosis. In: Endotext. South Dartmouth (MA): MDText.com, Inc. 2020.

[2] Wu AM, Bisignano C, James SL, Abady GG, Abedi A, Abu-Gharbieh E, et al. Global, regional, and national burden of bone fractures in 204 countries and territories, 1990–2019: a systematic analysis from the Global Burden of Disease Study 2019. Lancet Healthy Longev. 1990;2:e580. 10.1016/S2666-7568(21)00172-0. PMC8547262 34723233

[3] Sarafrazi N, Wambogo EA, Shepherd JA. Osteoporosis or low bone mass in older adults: United States, 2017–2018. NCHS Data Brief. 2021;405:1–8. 10.15620/CDC:103477 34029181

[4] Ogbodo JO, Agbo CP, Njoku UO, Ogugofor MO, Egba SI, Ihim SA, et al. Alzheimer’s Disease: Pathogenesis and Therapeutic Interventions. Curr Aging Sci. 2021;15:2–25. 10.2174/1874609814666210302085232 33653258

[5] 2023 Alzheimer’s disease facts and figures. Alzheimer’s & Dementia 2023;19:1598–695. 10.1002/ALZ.13016 36918389

[6] Nicholson WK, Silverstein M, Wong JB, Chelmow D, Coker TR, Davis EM, et al. Screening for Osteoporosis to Prevent Fractures: US Preventive Services Task Force Recommendation Statement. JAMA. 2025;333:498–508. 10.1001/JAMA.2024.27154 39808425

[7] Yu X, Sun S, Zhang S, Hao Q, Zhu B, Teng Y, et al. A pooled analysis of the association between sarcopenia and osteoporosis. Medicine (Baltimore). 2022;101:e31692. 10.1097/MD.0000000000031692. PMC9678526 36401390

[8] Amini N, Ibn Hach M, Lapauw L, Dupont J, Vercauteren L, Verschueren S, et al. Meta-analysis on the interrelationship between sarcopenia and mild cognitive impairment, Alzheimer’s disease and other forms of dementia. J Cachexia Sarcopenia Muscle. 2024;15:1240–53. 10.1002/JCSM.13485. PMC11294028 38715252

[9] Wang HS, Karnik SJ, Margetts TJ, Plotkin LI, Movila A, Fehrenbacher JC, et al. Mind Gaps and Bone Snaps: Exploring theConnection Between Alzheimer’s Disease and Osteoporosis. Curr Osteoporos Rep. 2024;22:483. 10.1007/S11914-023-00851-1 PMC11420299 38236512

[10] Margetts TJ, Wang HS, Karnik SJ, Plotkin LI, Movila A, Oblak AL, et al. From the mind to the spine: the intersecting world of Alzheimer’s and osteoporosis. Curr Osteoporos Rep. 2024;22:152–64. 10.1007/S11914-023-00848-W. PMC10912148 38334917

[11] Wanionok NE, Morel GR, Fernández JM. Osteoporosis and Alzheimer´s disease (or Alzheimer´s disease and Osteoporosis). Ageing Res Rev. 2024;99:102408. 10.1016/J.ARR.2024.102408 38969142

[12] Xiao T, Ghatan S, Mooldijk SS, Trajanoska K, Oei L, Gomez MM, et al. Association of Bone Mineral Density and Dementia The Rotterdam Study. Neurology. 2023;100:E2125–33. 10.1212/WNL.0000000000207220 PMC10186235 36948596

[13] Bliuc D, Tran T, Adachi JD, Atkins GJ, Berger C, van den Bergh J, et al. Cognitive decline is associated with an accelerated rate of bone loss and increased fracture risk in women: a prospective study from the Canadian Multicentre Osteoporosis Study. J Bone Miner Res. 2021;36:2106–15. 10.1002/JBMR.4402. 34289172

[14] Yuan J, Meloni BP, Shi T, Bonser A, Papadimitriou JM, Mastaglia FL, et al. The potential influence of bone-derived modulators on the progression of Alzheimer’s disease. J Alzheimer’s Disease. 2019;69:59–70. 10.3233/JAD-181249. 30932886

[15] Zhou R, Guo Q, Xiao Y, Guo Q, Huang Y, Li C, et al. Endocrine role of bone in the regulation of energy metabolism. Bone Res. 2021;19. 10.1038/s41413-021-00142-4. PMC8137703 34016950

[16] Khosla S. Evidence in humans for bone as an endocrine organ regulating energy metabolism. Curr Opin Endocr Metab Res. 2023;31:100471. 10.1016/J.COEMR.2023.100471. PMC10417886 37576432

[17] Oury F, Khrimian L, Denny CA, Gardin A, Chamouni A, Goeden N, et al. Maternal and offspring pools of osteocalcin influence brain development and functions. Cell. 2013;155:228–41. 10.1016/j.cell.2013.08.042. PMC3864001 24074871

[18] Khrimian L, Obri A, Ramos-Brossier M, Rousseaud A, Moriceau S, Nicot AS, et al. Gpr158 mediates osteocalcin’s regulation of cognition. J Exp Med. 2017;214:2859. 10.1084/JEM.20171320. PMC5626410 28851741

[19] Mosialou I, Shikhel S, Liu JM, Maurizi A, Luo N, He Z, et al. MC4R-dependent suppression of appetite by bone-derived lipocalin 2. Nature. 2017;543:385. 10.1038/NATURE21697. PMC5975642 28273060

[20] He T, Qin L, Chen S, Huo S, Li J, Zhang F, et al. Bone-derived factors mediate crosstalk between skeletal and extra-skeletal organs. Bone Res. 2025;13:49. 10.1038/S41413-025-00424-1. PMC12044029 40307216

[21] Yu J, Ji L, Liu Y, Wang X, Wang J, Liu C. Bone-brain interaction: mechanisms and potential intervention strategies of biomaterials. Bone Res. 2025;13:1–20. 10.1038/s41413-025-00404-5. PMC11914511 40097409

[22] Otto E, Knapstein PR, Jahn D, Appelt J, Frosch KH, Tsitsilonis S, et al. Crosstalk of brain and bone—clinical observations and their molecular bases. Int J Mol Sci. 2020;21:4946. 10.3390/IJMS21144946. PMC7404044 32668736

[23] Van Bezooijen RL, Ten Dijke P, Papapoulos SE, Löwik CWGM. SOST/sclerostin, an osteocyte-derived negative regulator of bone formation. Cytokine Growth Factor Rev. 2005;16:319–27. 10.1016/J.CYTOGFR.2005.02.005. 15869900

[24] Semënov M, Tamai K, He X. SOST is a ligand for LRP5/LRP6 and a Wnt signaling inhibitor. J Biol Chem. 2005;280:26770–5. 10.1074/jbc.M504308200. 15908424

[25] Kim J, Han W, Park T, Kim EJ, Bang I, Lee HS, et al. Sclerostin inhibits Wnt signaling through tandem interaction with two LRP6 ectodomains. Nat Commun. 2020. 10.1038/S41467-020-19155-4. PMC7585440 33097721

[26] Diegel CR, Kramer I, Moes C, Foxa GE, McDonald MJ, Madaj ZB, et al. Inhibiting WNT secretion reduces high bone mass caused by Sost loss-of-function or gain-of-function mutations in Lrp5. Bone Res. 2023;11:1–12. 10.1038/s41413-023-00278-5. PMC10447437 37612291

[27] Qian Z, Li H, Yang H, Yang Q, Lu Z, Wang L, et al. Osteocalcin attenuates oligodendrocyte differentiation and myelination via GPR37 signaling in the mouse brain. Sci Adv. 2021;7:eabi5811. 10.1126/SCIADV.ABI5811. PMC8535816 34678058

[28] Shao J, Wang Z, Yang T, Ying H, Zhang Y, Liu S. Bone regulates glucose metabolism as an endocrine organ through osteocalcin. Int J Endocrinol. 2015. 10.1155/2015/967673. PMC4383405 25873961

[29] Ferron M, Hinoi E, Karsenty G, Ducy P. Osteocalcin differentially regulates β cell and adipocyte gene expression and affects the development of metabolic diseases in wild-type mice. Proc Natl Acad Sci U S A. 2008;105:5266. 10.1073/PNAS.0711119105. PMC2278202 18362359

[30] Obri A, Khrimian L, Karsenty G, Oury F. Osteocalcin in the brain: from embryonic development to age-related decline in cognition. Nat Rev Endocrinol. 2018;14:174. 10.1038/NRENDO.2017.181. PMC5958904 29376523

[31] Hauschka PV, Lian JB, Gallop PM. Direct identification of the calcium-binding amino acid, gamma-carboxyglutamate, in mineralized tissue. Proc Natl Acad Sci U S A. 1975;72:3925–9. 10.1073/pnas.72.10.3925. PMC433109 1060074

[32] Lian JB, Hauschka PV, Gallop PM. Properties and biosynthesis of a vitamin K-dependent calcium binding protein in bone. Fed Proc. 1978;37:2615–20. 700171

[33] Price PA, Otsuka AA, Poser JW, Kristaponis J, Raman N. Characterization of a gamma-carboxyglutamic acid-containing protein from bone. Proc Natl Acad Sci U S A. 1976;73:1447–51. 10.1073/pnas.73.5.1447. PMC430313 1064018

[34] Price PA, Nishimoto SK. Radioimmunoassay for the vitamin K-dependent protein of bone and its discovery in plasma. Proc Natl Acad Sci U S A. 1980;77:2234–8. 10.1073/pnas.77.4.2234. PMC348687 6966406

[35] Ferron M, Wei J, Yoshizawa T, Del Fattore A, DePinho RA, Teti A, et al. Insulin signaling in osteoblasts integrates bone remodeling and energy metabolism. Cell. 2010;142:296–308. 10.1016/j.cell.2010.06.003. PMC2910411 20655470

[36] Shan C, Zhang D, Ma D, Hou Y, Zhuang Q, Gong Y, et al. Osteocalcin ameliorates cognitive dysfunctions in a mouse model of Alzheimer’s disease by reducing amyloid β burden and upregulating glycolysis in neuroglia. Cell Death Discov. 2023;9:46. 10.1038/S41420-023-01343-Y. PMC9902399 36746932

[37] Kjeldsen L, Johnsen AH, Sengeløv H, Borregaard N. Isolation and primary structure of NGAL, a novel protein associated with human neutrophil gelatinase. J Biol Chem. 1993;268:10425–32. 10.1016/S0021-9258(18)82217-7. 7683678

[38] Flo TH, Smith KD, Sato S, Rodriguez DJ, Holmes MA, Strong RK, et al. Lipocalin 2 mediates an innate immune response to bacterial infection by sequestrating iron. Nature. 2004;432:917–21. 10.1038/nature03104. 15531878

[39] Goetz DH, Holmes MA, Borregaard N, Bluhm ME, Raymond KN, Strong RK. The Neutrophil Lipocalin NGAL Is a Bacteriostatic Agent that Interferes with Siderophore-Mediated Iron Acquisition. Mol Cell. 2002;10:1033–43. 10.1016/S1097-2765(02)00708-6 12453412

[40] Wu BW, Guo JD, Wu MS, Liu Y, Lu M, Zhou YH, et al. Osteoblast-Derived Lipocalin-2 Regulated by miRNA-96-5p/Foxo1 Advances the Progression of Alzheimer’s Disease. Epigenomics. 2020;12:1501–13. 10.2217/EPI-2019-0215 32901506

[41] Eruysal E, Ravdin L, Kamel H, Iadecola C, Ishii M. Plasma lipocalin-2 levels in the preclinical stage of Alzheimer’s disease. Alzheimer’s Dementia: Diagnosis Assess Disease Monit. 2019;11:646. 10.1016/J.DADM.2019.07.004. PMC6733778 31517027

[42] Hermann P, Villar-Piqué A, Schmitz M, Schmidt C, Varges D, Goebel S, et al. Plasma lipocalin 2 in Alzheimer’s disease: potential utility in the differential diagnosis and relationship with other biomarkers. Alzheimers Res Ther. 2022;14:9. 10.1186/S13195-021-00955-9. PMC8759265 35027079

[43] Liu ZT, Liu MH, Xiong Y, Wang YJ, Bu XL. Crosstalk between bone and brain in Alzheimer’s disease: Mechanisms, applications, and perspectives. Alzheimer’s & Dementia. 2024;20:5720–39. 10.1002/ALZ.13864. PMC11350061 38824621

[44] Qi XS, He X, Peng Y, He XH, Yang QY, Jiao K, et al. Roles of osteocalcin in the central nervous system. CNS Neurosci Ther. 2024;30:e70016. 10.1111/CNS.70016. PMC11386255 39252492

[45] Li X, Wang X, Guo L, Wu K, Wang L, Rao L, et al. Association between lipocalin-2 and mild cognitive impairment or dementia: A systematic review and meta-analysis of population-based evidence. Ageing Res Rev. 2023;89:101984. 10.1016/J.ARR.2023.101984. 37330019

[46] Li F. Alzheimer’s disease increases the incidence of hospitalization due to fall-related bone fracture in elderly Chinese. Int J Gerontol. 2016;10:227–31. 10.1016/J.IJGE.2015.09.003.

[47] Liu D, Zhou H, Tao Y, Tan J, Chen L, Huang H, et al. Alzheimer’s disease is associated with increased risk of osteoporosis: the Chongqing aging study. Curr Alzheimer Res. 2016;13:1165–72. 10.2174/15672050113109990149. 23919776

[48] Zhao Y, Shen L, Ji HF. Alzheimer’s disease and risk of hip fracture: a meta-analysis study. Sci World J. 2012. 10.1100/2012/872173. PMC3354728 22629218

[49] Kostev K, Hadji P, Jacob L. Impact of Osteoporosis on the Risk of Dementia in Almost 60,000 Patients Followed in General Practices in Germany. J Alzheimers Dis. 2018;65:401–7. 10.3233/JAD-180569 30056429

[50] Kang HG, Park HY, Ryu HU, Suk SH. Bone mineral loss and cognitive impairment: the PRESENT project. Medicine (Baltimore). 2018. 10.1097/MD.0000000000012755. PMC6203493 30313083

[51] Kwon MJ, Kim JH, Kim JH, Cho SJ, Nam ES, Choi HG. The occurrence of Alzheimer’s disease and Parkinson’s disease in individuals with osteoporosis: a longitudinal follow-up study using a national health screening database in Korea. Front Aging Neurosci. 2021;13:786337. 10.3389/FNAGI.2021.786337. PMC8692765 34955816

[52] Zhou R, Zhou H, Rui L, Xu J. Bone loss and osteoporosis are associated with conversion from mild cognitive impairment to Alzheimer’s disease. Curr Alzheimer Res. 2014;11:706–13. 10.2174/1567205011666140812115818. 25115539

[53] Lu J, Mastaglia F, Tai ACP, Bulsara MK, Blakeney WG, Inderjeeth CA, et al. Association of heel bone mineral density with incident dementia among ageing adults: a population-based study from the UK Biobank. Aging Clin Exp Res. 2025;37:1–10. 10.1007/S40520-025-03100-W. PMC12267336 40668308

[54] Hu H, Mei J, Cai Y, Ding H, Niu S, Zhang W, et al. No genetic causal association between Alzheimer’s disease and osteoporosis: a bidirectional two-sample Mendelian randomization study. Front Aging Neurosci. 2023;15:1090223. 10.3389/FNAGI.2023.1090223. PMC9905740 36761181

[55] Dengler-Crish CM, Ball HC, Lin L, Novak KM, Cooper LN. Evidence of Wnt/β-catenin alterations in brain and bone of a tauopathy mouse model of Alzheimer’s disease. Neurobiol Aging. 2018;67:148–58. 10.1016/J.NEUROBIOLAGING.2018.03.021. 29660685

[56] Nagarajan A, Laird J, Ugochukwu O, Reppe S, Gautvik K, Ross RD, et al. Network Analysis of Brain and Bone Tissue Transcripts Reveals Shared Molecular Mechanisms Underlying Alzheimer’s Disease and Related Dementias and Osteoporosis. Journals Gerontology: Ser A. 2024;79:211. 10.1093/GERONA/GLAE211 PMC11503475 39194133

[57] Vlashi R, Zhang X, Wu M, Chen G. Wnt signaling: essential roles in osteoblast differentiation, bone metabolism and therapeutic implications for bone and skeletal disorders. Genes Dis. 2023;10:1291–317. 10.1016/J.GENDIS.2022.07.011. PMC10311035 37397540

[58] Kobayashi Y, Maeda K, Takahashi N. Roles of Wnt signaling in bone formation and resorption. Japanese Dent Sci Rev. 2008;44:76–82. 10.1016/J.JDSR.2007.11.002.

[59] Zhang C, Cho K, Huang Y, Lyons JP, Zhou X, Sinha K, et al. Inhibition of Wnt signaling by the osteoblast-specific transcription factor Osterix. Proc Natl Acad Sci U S A. 2008;105:6936–41. 10.1073/PNAS.0710831105. PMC2383965 18458345

[60] Li X, Zhang Y, Kang H, Liu W, Liu P, Zhang J, et al. Sclerostin binds to LRP5/6 and antagonizes canonical Wnt signaling. J Biol Chem. 2005;280:19883–7. 10.1074/jbc.M413274200. 15778503

[61] Delgado-Calle J, Pérez-Campo FM, Riancho JA. Advances in the study of the mechanisms involved in the modulation of the expression of sclerostin in human cells. Revista de Osteoporos y Metabolismo Mineral. 2014;6:103–8. 10.4321/S1889-836X2014000400006.

[62] McClung MR. Sclerostin antibodies in osteoporosis: latest evidence and therapeutic potential. Ther Adv Musculoskelet Dis. 2017;9:263–70. 10.1177/1759720X17726744 PMC5613857 28974988

[63] Drake MT, Khosla S. Hormonal and systemic regulation of sclerostin. Bone. 2017;96:8–17. 10.1016/J.BONE.2016.12.004. PMC5329134 27965160

[64] Spatz JM, Wein MN, Gooi JH, Qu Y, Garr JL, Liu S, et al. The Wnt inhibitor sclerostin is up-regulated by mechanical unloading in osteocytes in vitro. J Biol Chem. 2015;290:16744–58. 10.1074/JBC.M114.628313. PMC4505423 25953900

[65] Ardawi MSM, Al-Kadi HA, Rouzi AA, Qari MH. Determinants of serum sclerostin in healthy pre- and postmenopausal women. J Bone Miner Res. 2011;26:2812–22. 10.1002/JBMR.479. 21812027

[66] Hampel H, Hardy J, Blennow K, Chen C, Perry G, Kim SH, et al. The Amyloid-β Pathway in Alzheimer’s Disease. Mol Psychiatry. 2021;26:10. 10.1038/s41380-021-01249-0 PMC8758495 34456336

[67] Yuan J, Pedrini S, Thota R, Doecke J, Chatterjee P, Sohrabi HR, et al. Elevated plasma sclerostin is associated with high brain amyloid-β load in cognitively normal older adults. NPJ Aging. 2023. 10.1038/s41514-023-00114-4. PMC10477312 37666862

[68] Dicarlo M, Pignataro P, Urso D, Zecca C, Dell’Abate MT, Borlizzi F, et al. Cerebrospinal fluid sclerostin levels in the early Alzheimer’s disease stages. Alzheimer’s Dementia: Diagnosis Assess Disease Monit. 2026;18:e70297. 10.1002/dad2.70297. PMC12976974 41821749

[69] Pignataro P, Dicarlo M, Urso D, Zecca C, Colaianni G, Dell’Abate MT, et al. Elevated sclerostin levels in cerebrospinal fluid are associated with cognitive impairment in the Alzheimer’s disease continuum. Alzheimer’s Dementia: Diagnosis Assess Disease Monit. 2026;18:e70417. 10.1002/dad2.70417. PMC13319414 42388896

[70] Inestrosa NC, Varela-Nallar L. Wnt signaling in the nervous system and in Alzheimer’s disease. J Mol Cell Biol. 2014;6:64–74. 10.1093/JMCB/MJT051. 24549157

[71] Inestrosa NC, De Ferrari GV, Garrido JL, Alvarez A, Olivares GH, Barría MI, et al. Wnt signaling involvement in β-amyloid-dependent neurodegeneration. Neurochem Int. 2002;41:341–4. 10.1016/S0197-0186(02)00056-6. 12176076

[72] Liu CC, Tsai CW, Deak F, Rogers J, Penuliar M, Sung YM, et al. Deficiency in LRP6-Mediated Wnt Signaling Contributes to Synaptic Abnormalities and Amyloid Pathology in Alzheimer’s Disease. Neuron. 2014;84:63–77. 10.1016/J.NEURON.2014.08.048 PMC4199382 25242217

[73] Toledo EM, Inestrosa NC. Activation of Wnt signaling by lithium and rosiglitazone reduced spatial memory impairment and neurodegeneration in brains of an APPswe/PSEN1ΔE9 mouse model of Alzheimer’s disease. Mol Psychiatry. 2010;15(3):272–85. 10.1038/mp.2009.72. 19621015

[74] Scali C, Caraci F, Gianfriddo M, Diodato E, Roncarati R, Pollio G, et al. Inhibition of Wnt signaling, modulation of Tau phosphorylation and induction of neuronal cell death by DKK1. Neurobiol Dis. 2006;24:254–65. 10.1016/J.NBD.2006.06.016. 16919965

[75] Aghaizu ND, Jin H, Whiting PJ. Dysregulated Wnt Signalling in the Alzheimer’s Brain. Brain Sci. 2020;10:902. 10.3390/BRAINSCI10120902 PMC7761504 33255414

[76] Purro SA, Dickins EM, Salinas PC. The Secreted Wnt Antagonist Dickkopf-1 Is Required for Amyloid β-Mediated Synaptic Loss. J Neurosci. 2012;32:3492–8. 10.1523/JNEUROSCI.4562-11.2012 PMC6621056 22399772

[77] Marzo A, Galli S, Lopes D, McLeod F, Podpolny M, Segovia-Roldan M, et al. Reversal of synapse degeneration by restoring Wnt signaling in the adult hippocampus. Curr Biol. 2016;26:2551–61. 10.1016/J.CUB.2016.07.024. PMC5070786 27593374

[78] Guo X, Tang P, Liu P, Liu Y, Chong L, Li R. Dkk1: A promising molecule to connect Alzheimer’s disease and osteoporosis. Med Hypotheses. 2016;88:30–2. 10.1016/J.MEHY.2015.12.023. 26880631

[79] Shi T, Shen S, Shi Y, Wang Q, Zhang G, Lin J, et al. Osteocyte-derived sclerostin impairs cognitive function during ageing and Alzheimer’s disease progression. Nat Metab. 2024;6:531–49. 10.1038/s42255-024-00989-x. 38409606

[80] Gawdi R, Shumway KR, Emmady PD. Physiology, Blood Brain Barrier. [Updated 2023 Mar 17]. In: StatPearls [Internet]. Treasure Island (FL): StatPearls Publishing. 2023. https://www.ncbi.nlm.nih.gov/books/NBK557721/32491653

[81] Walker J, editor. The Proteomics Protocols Handbook. 1st ed. NJ: Humana Totowa. 2005. 10.1385/1592598900

[82] Fong CW. Permeability of the Blood–Brain Barrier: Molecular Mechanism of Transport of Drugs and Physiologically Important Compounds. J Membr Biol. 2015;248:651–69. 10.1007/S00232-015-9778-9/TABLES/2 25675910

[83] Jia L, Piña-Crespo J, Li Y. Restoring Wnt/β-catenin signaling is a promising therapeutic strategy for Alzheimer’s disease. Mol Brain. 2019. 10.1186/S13041-019-0525-5. PMC6894260 31801553

[84] Sebastian A, Loots GG. Genetics of Sost/SOST in sclerosteosis and van Buchem disease animal models. Metabolism. 2018;80:38–47. 10.1016/J.METABOL.2017.10.005. 29080811

[85] Baron R, Gori F. Targeting WNT signaling in the treatment of osteoporosis. Curr Opin Pharmacol. 2018;40:134–41. 10.1016/J.COPH.2018.04.011. 29753194

[86] Inokuchi S, Shimamoto K. Wnt/β-catenin pathway as a potential target for Parkinson’s disease: a cohort study of romosozumab using routinely collected health data in Japan. Front Pharmacol. 2024;15:1411285. 10.3389/FPHAR.2024.1411285. PMC11298754 39104397

[87] Zhou Y, Lin J, Shao J, Zuo Q, Wang S, Wolff A, et al. Aberrant activation of Wnt signaling pathway altered osteocyte mineralization. Bone. 2019;127:324–33. 10.1016/J.BONE.2019.06.027. 31260814

[88] Wang Z, Zhao T, Zhang S, Wang J, Chen Y, Zhao H, et al. The Wnt signaling pathway in tumorigenesis, pharmacological targets, and drug development for cancer therapy. Biomark Res. 2021;9:1–16. 10.1186/S40364-021-00323-7. PMC8422786 34488905

[89] Ho KH, Trapp M, Guida C, Ivanova EL, De Jaime-Soguero A, Jabali A, et al. Activation of Wnt/β-catenin signaling is critical for the tumorigenesis of choroid plexus. Neuro Oncol. 2025;27:106–22. 10.1093/NEUONC/NOAE176. PMC11726344 39215664

